# Healing rate of macular edema secondary to branch retinal vein occlusion in two years after initiation of intravitreal ranibizumab later combined with other treatment as needed and characteristics of refractory cases

**DOI:** 10.1371/journal.pone.0278968

**Published:** 2023-01-03

**Authors:** Setsuko Kawakami, Yoshihiro Wakabayashi, Yoko Watanabe, Kazuhiko Umazume, Kaori Yamamoto, Hiroshi Goto

**Affiliations:** Department of Ophthalmology, Tokyo Medical University, Tokyo, Japan; Tsukazaki Hospital, JAPAN

## Abstract

**Purpose:**

To investigate the 2-year healing rate of macular edema (ME) secondary to branch retinal vein occlusion (BRVO) treated initially with intravitreal ranibizumab (IVR) and later combined with other treatment as needed, and the characteristics of refractory cases.

**Methods:**

130 patients (130 eyes) with BRVO-ME who received IVR initially were studied. Anti-vascular endothelial growth factor drug was additionally administered when ME relapsed or persisted. Photocoagulation was performed when the non-perfusion area (NPA) was ≥5 disc diameter (DD), and/or when ME relapsed due to microaneurysm. Patients were classified into a healed group [ME resolved in <2 years or mild ME remained without best-corrected visual acuity (BCVA) loss for ≥6 months] or refractory group (ME persisted for ≥2 years).

**Results:**

110 eyes were classified into the healed group, and 20 eyes into the refractory group. The healed group and refractory group had, respectively, mean follow-up periods of 21.2 and 37.4 months, and frequencies of NPA ≥5 DD of 55.5 and 25.0% (p = 0.015). In the healed group, mean BCVA (logMAR) improved significantly compared to baseline in all the periods until 24 months after treatment initiation and at the last visit (p<0.001). In the refractory group, mean BCVA improved significantly compared to baseline until 12 months after treatment initiation (p<0.05 for all periods), but was not significantly different at 18 or 24 months or at the last visit.

**Conclusion:**

In patients with BRVO-ME treated initially with IVR and later given additional treatments as needed, the healing rate was 84.6%. In eyes that healed within 2 years, BCVA improved relative to baseline throughout 24 months and at the last visit. In refractory eyes, BCVA improved only until 12 months, and thereafter deteriorated to baseline level at the last examination.

## Introduction

Macular edema (ME) is the main cause of vision loss in patients with branch retinal vein occlusion (BRVO). Although anti-vascular endothelial growth factor (VEGF) therapy is effective for the treatment of ME secondary to BRVO (BRVO-ME), repeated relapses and a protracted course are seen in some patients. In cases of repeated ME relapses, combined use of anti-VEGF therapy with retinal photocoagulation and/or switching to other anti-VEGF drugs or corticosteroids are common treatment options in clinical practice [1]. Treatment of BRVO requires consideration not only for ME but also for vitreous hemorrhage from retinal neovascularization. Photocoagulation has been reported to be effective in reducing the occurrence of retinal neovascularization and vitreous hemorrhage, and photocoagulation should be co-administered as appropriate in the treatment of BRVO [2].

To clarify the long-term outcome of BRVO-ME in real-world clinical practice, we examined the clinical courses of cases in which intravitreal ranibizumab injection (IVR) was initiated and later combined with photocoagulation and other treatments as needed, and investigated the characteristics of cases in which ME did not resolve after continuous treatment for more than 2 years.

## Patients and methods

This study was a retrospective case series of patients with BRVO-ME, in whom IVR was given as the first treatment at Tokyo Medical University Hospital between September 2013 and August 2018. The study adhered to the tenets of the Declaration of Helsinki and was approved by the institutional review board (approval number: SH3782). All subjects provided written informed consent. Eyes with other retinal disorders, and eyes with a history of intravitreal injection of anti-VEGF drug, intravitreal or sub-Tenon’s injection of triamcinolone acetonide, retinal photocoagulation, or vitreous surgery were excluded. All patients underwent ophthalmic examination before the first IVR and after IVR on monthly basis. Ophthalmic examination included best corrected visual acuity (BCVA) measurement, slit-lamp biomicroscopy, fundus examination, and optical coherence tomography (OCT). For a patient with new or persistent ME, additional IVR was given as needed (pro re nata: PRN), regardless of the foveal thickness (FT) measurement. However, if the patient did not wish to be re-injected and his/her vision remained unchanged, we did not inject even if ME was present, and continued the follow-up. When the response to IVR was absent or diminished or when ME relapsed frequently, the anti-VEGF drug was switched to aflibercept in some cases at the physician’s discretion. After retinal hemorrhage was reduced, fluorescein angiography (FA) was performed. Scatter photocoagulation was done when the non-perfusion area (NPA) was 5 disc diameter (DD) or greater, and photocoagulation was also performed for microaneurysm when ME relapsed more than 1 year after initiation of IVR.

The patients were classified into two groups. Patients in whom ME resolved in less than 2 years after the first IVR and did not recur for more than 6 months after the last anti-VEGF drug injection, or mild ME remained without visual acuity loss for more than 6 months from the final anti-VEGF drug injection were classified into the healed group (treatment completed group). Patients who had persistent or relapsed ME within 6 months after receiving anti-VEGF vitreous injection for more than 2 years following the initial IVR were classified in the refractory group (treatment not completed).

We compared the BCVA and FT at baseline as well as clinical background between the two groups. In each group, we examined the changes in BCVA and FT over the course of treatment relative to baseline. Visual acuity was measured using decimal visual acuity chart and converted to logarithm of the minimal angle resolution (logMAR) for statistical analysis. FT was measured using Zeiss Cirrus OCT (Carl Zeiss Meditec, Inc.) or DRI OCT Triton (Topcon, Inc.) as the average retinal thickness in the area centered on the fovea within a diameter of 1 mm determined automatically with the caliper measurement tool embedded in the OCT system.

Statistical analysis was performed using IBM SPSS Statistics Software version 28.0. Wilcoxon signed rank sum test, Mann-Whitney U test, and Fisher’s exact test were used in statistical analyses. Changes in BCVA or FT were assessed using one-way repeated measures analysis of variance (ANOVA) with mixed model followed by post hoc Bonferroni correction. A P value < 0.05 was considered statistically significant.

## Results

During the study period, 283 eyes of 280 patients with BRVO-ME started IVR treatment. Among these patients, 130 patients (131 eyes) who met the selection criteria and had data for the pre-determined analyses were included in the study. One patient had bilateral disease, and only the eye that had earlier onset was studied. Hence, the present study was conducted on 130 eyes of 130 patients. The clinical characteristics of healed and refractory patients are listed in Table 1. Of 130 eyes, 110 eyes (84.6%) were classified as healed and 20 eyes (15.4%) as refractory. In the healed group, the clinical courses of ME resolution after initiation of IVR were as follows: ME resolved in less than 1 year in 88 eyes (67.7%) and from 1 year to less than 2 years in 18 eyes (13.8%) without recurrence for more than 6 months from the last injection, while mild ME remained but without visual acuity loss for more than 6 months in 4 eyes (3.1%). There were no differences in baseline characteristics between the healed and refractory groups. Within 2 years after initiation of IVR, the proportion of patients with NPA of 5 DD or greater was significantly higher in the healed group (55.5% healed vs. 25.0% refractory; p = 0.015). The proportion of patients with leaky microaneurysm was higher in the refractory group (30.0% refractory vs 11.8% healed), but the difference was not significant (Table 1).

**Table 1:** Characteristics of patients in healed group and refractory group.

	Healed group	Refractory group	*P-value* (Healed vs Refractory)
Number of patients	110	20	
Men/women	51/59	5/15	0.092
Number of eyes	110	20	
**Baseline**			
Age, years; mean±SD	63.7±11.6	69.6±10.6	0.060
(range)	(29–89)	(54–88)	
General condition, no. of patients			
Hypertension	81	13	0.426
Hyperlipidaemia	9	4	0.217
Diabetes	10	0	0.359
Lens status: phakia/IOL, no. of eyes	98/12	16/4	0.270
BRVO location: upper/lower, no. of eyes	66 /44	10/10	0.464
Bleeding: not limited/limited to macula, no. of eyes	91/20	17/3	1.000
BCVA, logMAR	0.36±0.34	0.31±0.23	0.726
(decimal)	(0.02–1.5)	(0.1–1.2)	
Foveal thickness, μm; mean±SD	489±160	478±121	0.931
(range)	(227–1148)	(261–825)	
Serous RD, no. of eyes (%)	32	4	0.588
Foveal serous RD height, μm; mean±SD	64±12	30±76	0.343
(range)	(0–723)	(0–306)	
**Findings within 2 years of initial IVR**			
Non-perfusion area ≥5 DD, no. of eyes (%)	61 (55.5%)	5 (25.0%)	0.015
Microaneurysm, no. of eyes (%)	13 (11.8%)	6 (30.0%)	0.077

*IOL;* intraocular lens, *BRVO;* branch retinal vein occlusion, *BCVA;* best corrected visual acuity, *DD*; disc diameter, *IVR*; intravitreal injection of ranibizumab, *logMAR;* logarithm of minimal angle resolution, *RD;* retinal detachment, *SD;* standard deviation

The mean follow-up period was 21.2±12.1 months in the healed group and 37.4±14.8 months in the refractory group. The number of intravitreal anti-VEGF injections received per patient until the final examination was 2.9±1.9 in the healed group and 9.8±4.1 in the refractory group. Nine (8.2%) eyes in the healed group and 10 (50.0%) eyes in the refractory group were switched to aflibercept.

**Fig 1:**
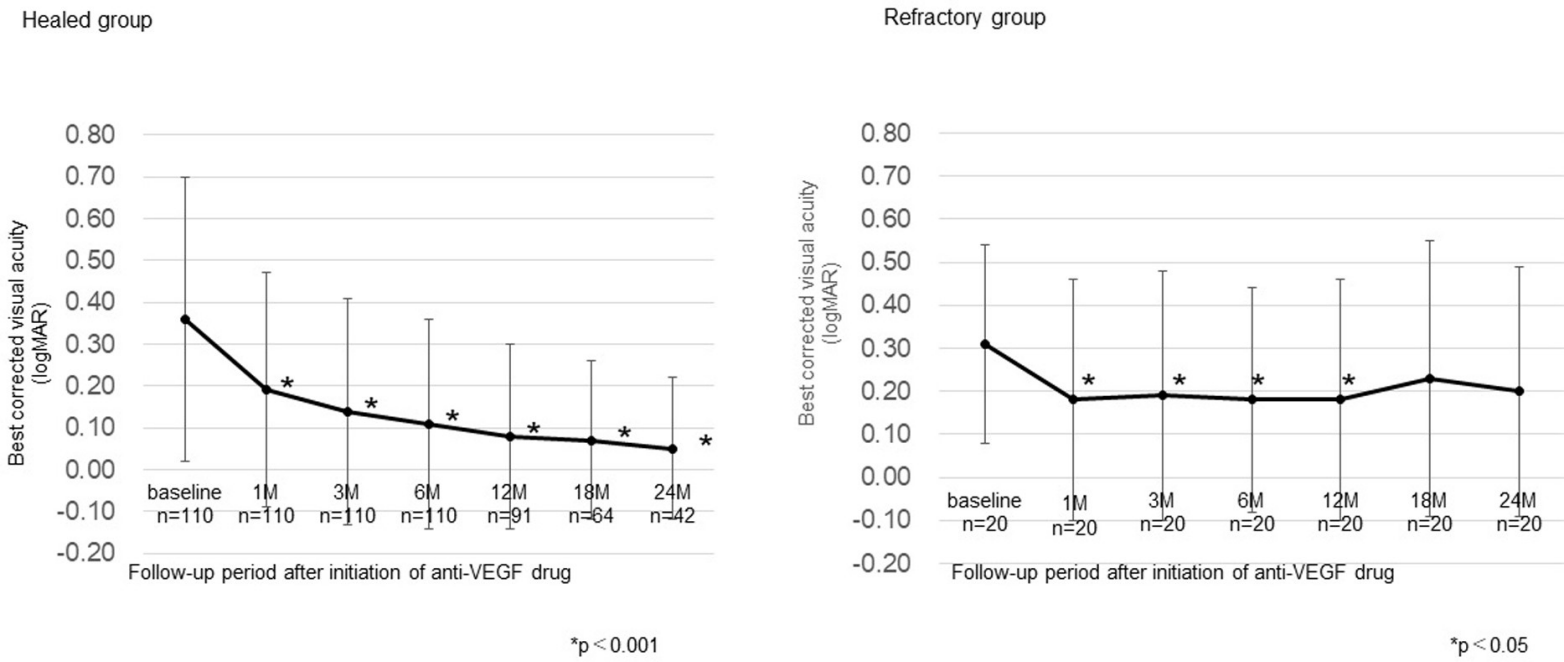
Changes in best corrected visual acuity over the course of treatment until 24 months. In the healed group, best corrected visual acuity (BCVA) improved significantly compared to baseline throughout the treatment period until 24 months. In the refractory group, BCVA improved significantly relative to baseline until 12 months after the start of treatment, and was not significantly different at 18 or 24 months. Data are expressed as mean ± standard deviation.

Fig 1 shows the changes in mean BCVA over the clinical course until 24 months, and Table 2 shows BCVA at the final examination in the healed and refractory groups. In the healed group, the mean BCVA (logMAR) improved significantly in all the periods after treatment initiation and at the final examination compared to baseline (p<0.001). In the refractory group, the mean BCVA improved significantly from baseline until 12 months after the start of treatment (all periods: p<0.05), and was not significantly different from baseline at 18 or 24 months, or at the final examination.

**Table 2:** Changes in best corrected visual acuity and foveal thickness at final examination.

	Healed group	*P-value*	Refractory group	*P-value*
		vs baseline		vs baseline
Follow-up, months	21.2±12.1		37.4±14.8	
(range)	(6–63)		(24–77)	
Best corrected visual acuity, logMAR				
Baseline	0.36±0.34		0.31±0.23	
Final	0.05±0.22	<0.001	0.25±0.32	0.214
Foveal thickness, μm				
Baseline	489±160		478±121	
Final	255±39	<0.001	352±122	0.010

Data are expressed as mean ± standard deviation (range)

**Fig 2:**
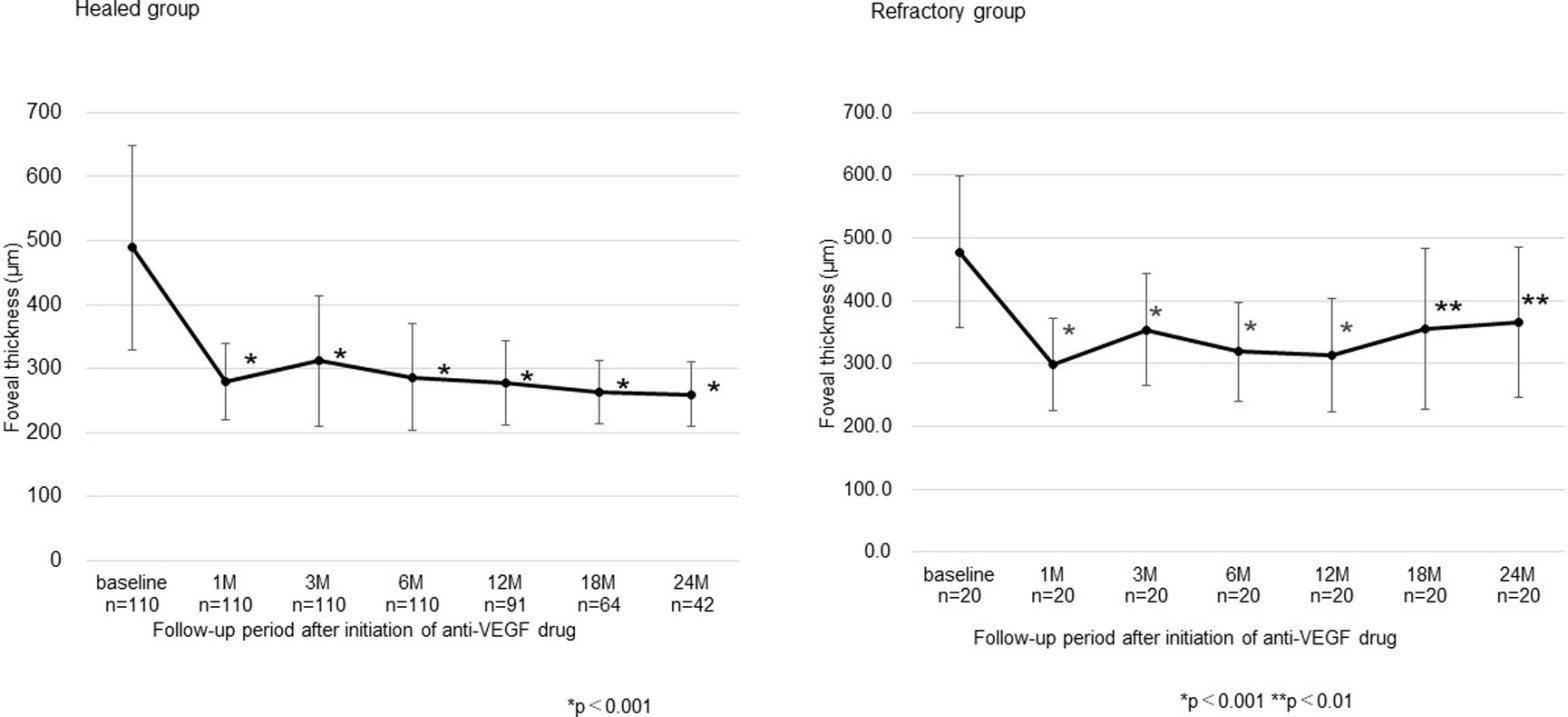
Changes in foveal thickness. Foveal thickness decreased significantly compared to baseline in all the periods until 24 months in both groups. Data are expressed as mean ± standard deviation.

Fig 2 shows the changes in mean FT over the clinical course until 24 months, and Table 2 shows FT at the final examination in the healed and refractory groups. In the healed group, the mean FT decreased significantly compared to baseline in all the periods (all periods, p<0.001). In the refractory group, the mean FT decreased significantly compared to baseline in all the periods (until 12 months, p<0.001; at 18 and 24 months, p<0.01; at the final examination, p = 0.010).

## Discussion

In this study, the healing rate of ME in patients with BRVO-ME treated initially with IVR was 84.6%. Excluding the subgroup with residual mild ME but no visual acuity loss, the ME resolution rate within 2 years after IVR initiation was 81.5%; comprising 67.7% within 1 year, and 13.8% from 1 year to less than 2 years. The corresponding rates in a previous report were 77%, 53%, and 24% [3], showing comparable ME resolution rates at 2 years to those in the present study.

In the healed group, BCVA improved significantly compared with baseline throughout the 2-year period after IVR initiation and also at the final observation. In the refractory group, although BCVA improved significantly compared with baseline until 12 months after starting IVR, visual acuity deteriorated at 18 and 24 months and at the final examination, and was not significantly different from baseline. Although FT decreased significantly compared with baseline after treatment initiation until the final examination in both the healing group and the refractory group, the visual outcome was less favorable in the refractory group. The refractory cases had a longer follow-up period and the macula was exposed to edema for a longer period of time than the healed cases, suggesting that long-term ME may affect the final visual acuity.

The healed group differed from the refractory group in one clinical variables. The proportion of patients who had NPA of 5 DD or greater was higher in the healed group than in the refractory group. Macular perfusion status was reported to affect the persistence of ME [4, 5], and cases with OCT angiographic finding of decreased vessel density in the macular region after anti-VEGF therapy was associated with more frequent ME recurrence and less visual acuity improvement [6]. Previous report suggests that NPA outside the macular region may not be involved in macular function [7]. It is unknown why ischemic BRVO was more common in the healed group in this study. In addition, whether administration of scatter photocoagulation to NPA improved ME in this study remains unclear, because there was no comparison with a group without photocoagulation under the same conditions. The RELATE trial found no additional benefit of scatter photocoagulation in improving ME [8]. Aiello et al. reported that photocoagulation reduced intraocular VEGF concentrations, which may influence the frequency of injections of anti-VEGF drugs [9].

The proportion of patients who had microaneurysm was higher in the refractory group than in the healed group, but the difference was not significant. Previous studies reported that microaneurysm was more common in recurrent ME cases [10–12]. While macular grid laser or photocoagulation for both NPA and edematous area with microaneurysm in the arcade has been shown to have no effective [13, 14], direct photocoagulation for microaneurysm has been found to be effective [10, 15]. Although whether direct photocoagulation for microaneurysm was effective in this study remains unclear because of the lack of a control group without photocoagulation under the same conditions, 13 of 19 eyes (68.4%) that received direct photocoagulation were healed. Direct photocoagulation for microaneurysm may be effective in some cases.

Ischemic BRVO has a risk of vitreous hemorrhage due to retinal neovascularization. In BRVO treatment, therefore, it is important not only to resolve ME but also to suppress vitreous hemorrhage due to neovascularization in the retina. In the Branch Vein Occlusion Study (BVOS), scatter photocoagulation reduced the incidence of neovascularization from 31% to 19% in ischemic BRVO with NPA greater than 5 DD, and the incidence of vitreous hemorrhage was reduced from 61% to 29% when neovascularization was already present [2]. The BVOS also showed that the incidence of vitreous hemorrhage was 12% when scatter photocoagulation was performed only before the development of neovascularization, whereas the incidence was 9% when scatter photocoagulation was performed only after neovascularization was detected. International guidelines recommend performing scatter photocoagulation for NPA of 5DD or more after, rather than before, the development of neovascularization due to concerns about the invasive nature of scatter photocoagulation, including visual field impairment and worsening of peripheral vision [16]. However, in real-world clinical practice, detection of neovascularization may be missed before vitreous hemorrhage occurs, especially in patients with health problems that impede the patients to attend regular follow-up visits [17]. In these patients, photocoagulation performed before neovascularization is detected may be effective in reducing the risk of future vitreous hemorrhage.

Limitations of this study include the single-institution and retrospective study design with a limited number of patients. Due to the lack of data about the time of BRVO-ME onset, we were unable to examine the effect of the duration from onset to treatment initiation on persistence or recurrence of ME. Because photocoagulation was performed in all the patients who had microaneurysm or NPA, it was impossible to compare photocoagulation with no photocoagulation.

In conclusion, in patients with BRVO-ME in whom IVR was initiated and later combined with photocoagulation or other treatment as needed, ME was healed in 84.6% of the patients within 2 years, resulting in treatment completion and improved BCVA accompanied by reduced FT. In patients with repeated relapses even after treatment for 2 years or longer, BCVA improved until 12 months and deteriorated thereafter to baseline level at the final examination.

## Supporting information

S1 File Database of all individuals. (XLSX)
